# The Public Health: Interviews with Local Authorities

**Published:** 1922-11

**Authors:** 


### THE PUBLIC HEALTH.

### INTERVIEWS WITH LOCAL AUTHORITIES.

### V.—NOTTINGHAM—AN INDUSTRIAL HEALTH RESORT.

AS we approached the City of Nottingham over the fine suspension bridge which spans the River Trent, and looked across the city to the slopes of Castle Hill, the question at once occurred to us : Where is the smoke ? We put this question to the Chairman of the Public Health Committee, Councillor Hutton, with whom was Dr. Boobbyer, the Medical Officer of Health, who has served the city so well for many years. We were at once informed that the manufacturers engaged in the staple industries of the city, lace and hosiery, especially the former, would not be at all tolerant of black smoke emerging from neighbours' chimneys ; that this strengthened the hands of the Medical Officer ; and, further, that the hilly formation of the city and the open country around tended to keep such smoke as there was well on the move. We find ourselves, therefore, in complete accord with the official handbook, which says that " the city has a remarkably clean appearance, such as one does not usually associate with a busy manufacturing centre, and a spaciousness, a dignity, and a suggestion of progress patent at once to every visitor."

The genial Chairman of the Public Health Committee looks forward to the city taking its place amongst the health resorts of the country. Some of the sick and maimed from other industrial areas already make their way to Nottingham in order to work under its more salubrious conditions. There is, indeed, nothing fantastic in the idea of Nottingham, setting a new fashion as an industrial centre, having a concurrent reputation as a health resort. At the same time, Nottingham would not have you disregard the facilities which she offers for industrial development, amongst which are to be included cheap sites, cheap fuel, abundant labour, and splendid railway and canal facilities. " To these may be added," says our friend, the official handbook, " the advantage of an environment that cannot fail to react favourably upon the workers and thus stimulate contentment and efficiency in their labour."

When we were surveying the city from the Castle, which occupies a magnificent position and contains the city's art gallery and museum, we were informed by a friend, who was also guide and philosopher, that he had found the air in Nottingham compare very favourably with the East coast air which he had been breathing for some 40 years previously. This is first-hand testimony, which is deserving of record.

### Housing.

Mr. Hutton paid tribute to the fine performance of the Housing Committee, under the chairmanship of Mr. Councillor Crane, in the progress which they had made since the war in their housing programme.

In regard to one part, at least, of her housing schemes, Nottingham enjoys the almost unique distinction of having carried through a complete building programme. The Sherwood Estate, which we visited in the company of the Medical Officer of Health, is really a garden city. It was purchased by the Corporation in September, 1919, and now contains nearly 600 houses of picturesque design and grouping. The undulating character of the ground, its elevated position, and its dry, sandy subsoil have rendered it particularly suitable for development. The belts of fine old trees considerably helped the lay-out and design. It is interesting to notice that, from amongst 25 architects who submitted designs for this scheme, the one who was appointed as the most successful competitor was a local architect, Mr. W. A. Kneller. The great majority of the houses are of parlour type, with three bedrooms, and the average cost somewhere about £900. It has sometimes been objected that tenants of the houses have not been drawn entirely from the classes which were most in need of the houses; but it must in any case be recognised that the development of the property is a clear addition to the housing accommodation of the city, and that it adds considerably to its amenities.

Altogether, in the city some 1,400 houses have been erected towards an estimated requirement of 3,500, and this is a proportion of the whole housing provision which a good many other areas will regard with envy and admiration. From a recent article in the local press we learn that small houses are now being erected in the Meadows District at a cost of some £300 each (this has been quoted elsewhere as an example of what can now be done), and that in a short time the work of clearing a portion of the Narrow Marsh will be begun. As this, however, will necessarily take time, it is gratifying to be able to say that very necessary sanitary improvements have been, and are being, effected meanwhile in the poorer quarters of the town.

### The Battle for Water-carriage.

"Since those bad old days," says the guide-book, referring particularly to an occasion (in 1831) when "a mob of politically-mad and drink-distraught citizens" had a difference of opinion with the Duke of Newcastle, and reduced his palace on Castle Hill to a smoking ruin, "the civic life of Nottingham has, happily, run in the paths of peace, being no longer hampered by such untoward happenings."

There are parts of the city to which, after due notice to the tenants, of course—and, incidentally, provision for their accommodation—this sort of treatment might be applied with advantage to the vital statistics of the city. The death-rate at the present time is about 11·5 per 1,000, and the infantile mortality rate below 80. These figures, though relatively good, are adversely affected by the existence of some slum areas, which would receive "honourable mention" in a worst-slum competition. It is up to Nottingham to get this blot off its escutcheon, and it is therefore satisfactory that, as above stated, the work of clearance of the worst area is shortly to be begun. The immediate effect of the foregoing quotation is, however, to indicate that—so far, at any rate, as hygienic peace is concerned—the progress in the matter of the conversion of parts of the city from the pail-closet system (or lack of system) to water-carriage has been not at all an unhampered advance. There were two main difficulties. As to the first, it is well to be frank. The City Fathers were apathetic. For years, Dr. Boobbyer's voice was that of one crying in the wilderness. His protestations were possibly regarded as the "vapourings of a visionary." His statistics of the incidence of enteric fever and diarrhœa took quite a long time to penetrate. To take the cases of enteric: In 1900, 1 case in 92 houses with pail-closets, as compared with 1 in 407 houses with water-closets; in 1905, 1 case in 181, and 1 in 571 respectively; in 1909, out of 141 cases of enteric, 123 occurred in houses with pail-closets. Dr. Boobbyer's trump card was a map with two conspicuous clusters of black spots—very unpleasant things to look at—both adjacent to "sanitary" depots to which the night-soil of the pail-closets was taken. Conversion began, very slowly at first. The other difficulty to which reference has been made had to be surmounted. This was the engineering difficulty, arising from the fact that, whereas the old city within the walls is on sandstone, the part to the south, known as the Marsh, is of mud and sand; the shifting character of the soil, and the effect of subsidence on pipes after they had been laid, rendered the work of drainage a matter of considerable difficulty.

"Until the whole of the dry system is replaced by one of water carriage," wrote the Medical Officer in 1915, "the city must be held to be lacking and behind the times in one of the most essential particulars of modern sanitary administration, and especially liable to outbreaks of certain fever and other diarrhœal diseases." At that time the number of conversions was slow—about 4,000 only in five years—but to-day the Medical Officer is able to announce with satisfaction that the number of houses which remain with the old pail closets has been reduced from 36,000 in 1912 to 2,000. His satisfaction is shared by the Chairman of the Health Committee. The main part of this work has been accomplished during Councillor Hutton's term of office as Chairman, and this is particularly appropriate in view of his personal experiences in visiting the slum areas in years gone by, and his determination that when the opportunity came to him agents for the spread of enteric and other infective diseases of the intestines should be swept away.

THE CITY'S PATHOLOGICAL LABORATORY.

In a well-organised community, the attacking of disease at its source, such as the onslaughts on uncleanliness and faulty sanitation, is accompanied by good, sound, albeit unobtrusive, work in the laboratory. As to the former, it would be interesting to know how many of the citizens of Nottingham have any conception of the manifold activities of their Medical Officer and those associated with him, and of the conglomeration of subjects which come under review of the Public Health Committee. Our interview was interrupted, for example, by a discussion on some immediate problem connected with backyard fowl keeping—a small matter, it is true, but one which takes its place with subjects so diversified as Inspection of Foodstuffs, Supervision of Midwives, Distribution of Free Milk, Inspection of Slaughterhouses, of Drains, and of Cowsheds, the provision of Hostels for Mothers and Babies, of Sanatorium treatment for the Tuberculous, of Clinics for the Treatment of Venereal Disease.

As to the work of the Pathological Laboratory, let us look at some details. Nottingham is particularly well served in this respect. We have it on independent testimony that some really first-class work is being carried out at the Laboratory. In 1921, 23,715 specimens were examined as follows :—

| | |
|---|---|
| Examinations in connection with Venereal Diseases .. .. .. .. | 12,954 |
| Examinations in accordance with various Public Health Acts .. .. .. | 7,515 |
| Clinical pathological examinations .. | 3,246 |
| Total .. .. .. .. .. | 23,715 |

Two features are of particular interest in connection with this work. One is that the City do a good deal of examination for the surrounding authorities and doctors ; thus, of the 12,954 V.D. examinations, 1,444 were undertaken for medical practitioners outside Nottingham, 2,988 for the Notts County Council, 315 for the Lincoln City Corporation, 146 for the Lincoln (Lindsey) County Council, and 489 for the Grimsby Borough Council. The number of specimens examined under various Public Health Acts was, as stated, 7,515. It was much higher in 1920, when 10,157 such examinations were made ; the decrease in 1921 is almost entirely accounted for by the reduction in the number of swabs submitted for examination for traces of diphtheria.

The other feature to be specially noted is the increasing tendency among general practitioners and hospitals to carry out their own examinations when these are of a simple nature ; this is all to the good, but the effect is, of course, that the numbers falling to be examined by the City Laboratory represent an increasing average of complexity.

THE CONTROL OF THE MINISTRY OF HEALTH.

The question of the arrangements made at the pathological laboratory led to an interesting talk with Mr. Hutton and Dr. Boobbyer as to the relations of the Council with the Ministry of Health. At the time when the laboratory was set up a few years ago it would appear that there was an understanding with the Department that an Exchequer contribution would be available on the basis of the number of venereal disease specimens examined, and the arrangements with other Authorities have been entered into by the City Council on the strength of this understanding. The Department are now limiting the contribution to 75 per cent. of that part of the expenditure in connection with the laboratory which is assessed as appertaining to the time estimated to be required in connection with the examination of V.D. specimens. This amount has been assessed at 15/40ths. This departure from a basis which the Council has regarded as permanent is leading to vigorous protests on the part of Mr. Hutton and his Committee. We thereupon inquired whether the Local Authority regarded the detailed control by the Ministry of their expenditure on the grant-carrying services—Tuberculosis, Venereal Disease, Maternity and Child Welfare—as irksome. We were assured that this was not the case, and it was interesting to have an expression of the personal view of the Chairman of the Public Health Committee that he was averse from any block-grant system such as recommended by the Geddes Committee, which he conceived to be not in the public interest in the long run. In expenditure on health services it was, he felt, essential that the Local Authority should have the stimulus and support which was derived from the Exchequer contributions ; and although it might be true to some extent to say that, sooner or later, the same body of people substantially paid for the services, either as ratepayers or taxpayers, the burden in the latter case was not so immediately felt, and Local Authorities were far more likely to embark on essential expenditure on health services if they were not deterred by the fact that the whole cost would fall immediately on the rates. It was true that when local expenditure carried with it a proportionate contribution from the Exchequer, detailed control on the part of the Ministry of Health was an inevitable part of such a system. This, however, appeared to Mr. Hutton to be all to the good, and so long as the control was exercised with the tact and forbearance to which they had been used, he thought that it was altogether in the interest of wise expenditure on health services along sound lines of development.

### THE SPAHLINGER TREATMENT OF TUBERCULOSIS.

THE announcement that the British Red Cross Society has made an appropriation in order to enable M. Henri Spahlinger to produce his treatment for tuberculosis on a larger scale has aroused a good deal of interest. It is the intention of the Society that the preparation should be tried under scientific supervision. The Ministry of Health has agreed to co-operate in further inquiries. Dr. A. S. MacNalty, a medical officer of the Ministry, recently visited M. Spahlinger's Institute at Carouge, just outside Geneva. M. Spahlinger claims to have found a vaccine suitable for early cases as well as for advanced cases of tuberculosis that have received a preliminary course of serum treatment. But authoritative medical opinion so far has declined to support him.

